# A Novel Binder-Assisted Atmospheric Sintering Route for Silicon Carbide (SiC) Production

**DOI:** 10.1021/acsomega.6c03885

**Published:** 2026-07-27

**Authors:** Yasemin Gulce, Selda Topcu Sendogdular, Levent Sendogdular

**Affiliations:** 1 Department of Materials Science and Engineering, Graduate School of Natural and Applied Sciences, 52958 Erciyes University, Kayseri 38039, Turkey; 2 Department of Metallurgical and Material Engineering, Faculty of Engineering, 52958 Erciyes University, Kayseri 38039, Turkey

## Abstract

Silicon carbide (SiC) is a high-performance ceramic known for its exceptional thermal and mechanical stability, yet its conventional synthesis requires high temperatures and inert atmospheres, leading to substantial cost and energy demands. This study introduces a potentially sustainable binder-assisted atmospheric sintering technique for β-SiC formation from SiO_2_ and activated carbon, eliminating the need for vacuum or protective gas environments. Using a hybrid graphite–mullite insulation system with sacrificial carbon barriers, β-SiC was successfully synthesized at temperatures as low as 1350 °C. The hybrid insulation acts as a passive getter, creating a localized reducing atmosphere, while the UF resin residue creates a carbon skeleton that enhances particle necking. XRD analyses confirmed the presence of 3C-SiC as the dominant phase, while binder addition markedly enhanced densification and mechanical strength. Incorporating urea–formaldehyde resin achieved a compressive strength of 25.5 MPa after only 15 min of sintering at 1500 °C—representing a 4-fold improvement over binder-free samples. Furthermore, specific surface area measurements revealed that the resin-bonded samples achieved substantial microstructural densification (0.35 m^2^/g) by contributing to the reduction of accessible open pore networks. This work provides a potentially scalable and simplified atmospheric processing strategy for SiC production, showing that a fully atmospheric route combined with binder chemistry can yield improved mechanical performance in β-SiC ceramics at significantly reduced temperature and time.

## Graphical abstract

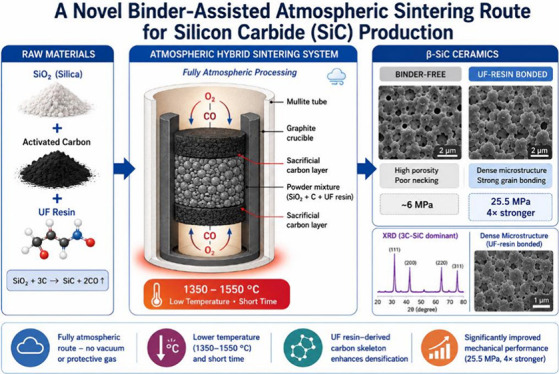

## 1 Introduction

Silicon carbide (SiC) is a high-performance ceramic material widely utilized in high-temperature, wear-resistant, corrosion-resistant, creep-resistant, and electronic applications due to its exceptional mechanical, thermal, and chemical properties. Its high hardness, low thermal expansion, and outstanding oxidation resistance make it indispensable in the aerospace, automotive, semiconductor, and energy industries. Furthermore, its excellent radiation resistance holds promise for nuclear applications.^1–5^

SiC is a unique ceramic material that exhibits one-dimensional polymorphism. While more than 250 polytypes exist, the cubic 3C (β-SiC) and hexagonal α-SiC (4H, 6H) are the most technologically relevant.^1,2^ While single-crystal SiC polytypes (4H, 6H) are dominant in high-voltage semiconductor electronics, polycrystalline SiC ceramics—the focus of this study—are critical for structural and thermal applications. Their high hardness, thermal shock resistance, and chemical inertness make them indispensable for abrasives, kiln furniture, and wear-resistant seals.^1^ Unlike the expensive growth of single crystals, the sintering of SiC powders targets cost-effective mass production.

The most commonly used method for producing SiC is the Acheson process, which involves a carbothermal reduction reaction between quartz (SiO_2_) and a carbon source at extremely high temperatures (>2000 °C) in electric furnaces.^6,7^ Although effective, this process requires expensive infrastructure, high energy consumption, and controlled atmospheres to prevent oxidation. Moreover, the elevated temperatures used in this process lead to substantial CO_2_ emissions, raising environmental concerns.^8,9^ Alternative techniques such as chemical vapor deposition (CVD) and sol–gel processing have been explored for the production of high-purity SiC; however, these methods often involve complex processing steps and high production costs.^10,12^

To address environmental and economic concerns, recent research has focused on developing low-temperature and sustainable alternatives. In this context, “low-temperature” refers to processes conducted below the conventional 2000–2400 °C range typically required for the Acheson process or pressureless sintering of pure SiC. The present study targets the 1350–1550 °C range to significantly reduce energy consumption while maintaining structural integrity. Techniques such as pressureless sintering under atmospheric conditions, self-propagating high-temperature synthesis (SHS), and magnesiothermic reduction have been investigated for their potential to lower processing temperatures and simplify equipment requirements.^13,14^

Perthuis and Colomban demonstrated that sol–gel-based chemical routes could significantly reduce the sintering temperatures of ceramic oxides due to the controlled chemistry of precursors and the sintering atmosphere. However, the sol–gel method still suffers from complex processing steps and low production volumes.^15^ Similarly, Ren et al. successfully produced porous SiC ceramics under low-temperature sintering environments using recycled silicon kerf waste, emphasizing both cost-effectiveness and material recyclability. Their method involved Ar, N_2_, and carbon-embedded atmospheres.^16^ Sizyakov et al. further investigated self-propagating synthesis mechanisms and demonstrated that β-SiC could be synthesized under relatively moderate thermal conditions and specific pressures.^17^ Hidayat et al. reported the production of β-SiC in an argon atmosphere using magnesiothermic reduction synthesis.^13^ Lavrenchuk et al. demonstrated the feasibility of synthesizing SiC under atmospheric conditions by utilizing an AC atmospheric-pressure arc reactor, where the reaction zone was regulated via CO/CO_2_ gas formation.^11^

Although previous studies have explored SiC formation under low-pressure, inert, or partially atmospheric environments, these approaches typically required protective gases (Ar, N_2_, and CO/CO_2_) or complex setups to suppress oxidation. Ren et al.^16^ produced porous SiC under Ar atmosphere with over 60% porosity, while Lavrenchuk et al.^11^ utilized an atmospheric-pressure arc reactor with CO/CO_2_ gas regulation. Mas’udah et al.^19^ synthesized β-SiC from SiO_2_ and activated carbon but reported limited densification due to oxidation.

In contrast, the present study proposes a fully atmospheric and binder-assisted sintering route that eliminates the need for controlled atmospheres. A hybrid graphite–mullite insulation system combined with sacrificial carbon barriers was developed to restrict oxidation, while binder chemistry was optimized to enhance microstructural integrity. This combination has not been systematically evaluated in prior studies and represents a potentially effective atmospheric processing approach to achieving β-SiC formation at significantly reduced temperature and duration. In this study, “sustainability” is defined by the elimination of the need for complex vacuum systems, continuous inert gas flows (e.g., argon), and the extreme energy demands (>2000 °C) typical of the Acheson process. By utilizing a passive hybrid graphite-mullite getter system, effective SiC synthesis is achieved at significantly lower temperatures under standard atmospheric conditions.

## 2 Materials and Methods

### 2.1 Sample Preparation

In this study, silicon carbide (SiC) was synthesized using SiO_2_ (quartz powder) and C (activated carbon) as raw materials. Quartz (SiO_2_) powder with an average particle size of 44 μm and activated carbon with an average particle size of 75 μm were used as the raw materials. To ensure close contact between particles during sintering, materials with similar particle sizes were selected.^17^ For the same purpose, the powders were mixed with a binder. In this work, poly­(vinyl alcohol) (PVA), which is commonly used as a binder in similar solid-state reactions in the literature, was employed.^19^ The ratio of raw materials in the mixture was set to 1:3 (SiO_2_:C), consistent with the Acheson process. The SiO_2_-C-PVA slurry was prepared with 35% water content, homogenized using a ball mill, and then dried in a laboratory oven until a 5% moisture level was reached.^20^ As reported by Freedman and Millard, wet processing methods involving slurry preparation and shaping provide more uniform particle distribution and lower porosity in SiC ceramics. Although dry pressing is faster, it poses a higher risk of density gradients and structural defects, especially in large-volume parts.^21^

The samples were shaped into cylindrical pellets under 150 MPa using a hydraulic press, based on similar studies.^16^ The sacrificial graphite plates used during sintering were also prepared by forming the same slurry, followed by drying and pressing.

To enable SiC synthesis under atmospheric conditions, a sintering kit was designed consisting of a graphite crucible (matched to the pellet size) nested within a mullite tube. The SiO_2_-C-PVA pellets and sacrificial graphite plates were placed inside the inner graphite crucible, which was inserted into the mullite tube. To minimize oxygen ingress through the top and bottom of the mullite–graphite assembly, sacrificial graphite plates and ceramic lids were used.

The sintering regime was designed by considering the transformation temperatures of quartz.^17^ The hybrid insulation system creates a localized reducing microenvironment essential for atmospheric sintering. As the sacrificial graphite crucible and barriers oxidize, they consume trapped oxygen, generating a CO-rich atmosphere (2C + O_2_ → 2CO) within the mullite tube. This ″passive getter″ mechanism maintains a partial pressure of oxygen low enough to stabilize SiC formation without external gas flow.^18^

To determine the optimum sintering conditions for SiC synthesis in atmospheric environments, sintering temperatures between 1350 and 1550 °C and durations ranging from 1 to 6 h were studied. The extended 6 h dwell time at elevated temperatures (1500–1550 °C) was selected to promote completion of the carbothermal reduction reaction and improve the crystallinity of the 3C-SiC phase. The complete experimental matrix, including the sample numbering, corresponding sintering temperatures, and holding times used throughout this study, is summarized in Table 1. This numbering system is consistently used in the subsequent characterization and discussion sections for clarity.

**Table 1:** Sintering Parameters of the Produced SiC Samples

sample no.	sintering temperature (°C)	sintering duration (h)
**1**	1350	1
**2**	1350	2
**3**	1350	3
**4**	1400	1
**5**	1400	2
**6**	1400	3
**7**	1500	1
**8**	1500	2
**9**	1500	3
**10**	1500	6
**11**	1550	6

In the subsequent phase of the study, it was concluded that the compressive strength results needed improvement. For this purpose, SiC powder previously produced via the hybrid sintering technique was mixed with a urea-formaldehyde (UF)-based resin, pressed, and then sintered. UF resin was specifically selected as a secondary binder due to its high char yield. Unlike PVA, which burns off almost completely, UF resin leaves a residual carbon skeleton upon decomposition. This residue bridges SiO_2_ and C particles, acting as a reactive sintering aid that enhances densification. The gradual thermal decomposition of the UF resin during heating is also believed to contribute to localized neck formation and enhanced interparticle bonding during atmospheric sintering. The mixing, moisture adjustment, and pressing steps of the sample preparation were performed in the same manner as in the earlier productions. During this stage, sintering was carried out in open atmospheric conditions at 1500 °C for 15 min. While designing the sintering regime, the precuring and decomposition temperatures of the UF resin were taken into consideration.

### 2.2 Characterization and Mechanical Testing

In order to determine the phase composition and crystalline structure of the samples, X-ray diffraction (XRD) analysis was conducted. The measurements were performed using Cu Kα radiation (λ = 1.5406 Å) in the 2θ range of 10–90°. The obtained diffractograms were analyzed to evaluate the presence of different polymorphs (3C, 6H) of β-SiC, as well as to identify possible secondary phases such as residual silica (SiO_2_) or carbon. Phase analyses were carried out using the HighScore software, setting the minimum significance value for each phase at 30. Phase identification was performed by comparing the diffraction patterns with the ICDD PDF database.^22^

Additionally, microstructural morphology and grain bonding were examined using scanning electron microscopy (SEM). SEM observations were performed at an accelerating voltage of 25 kV under high-vacuum conditions. Thermal behavior and oxidation resistance of the samples were evaluated via differential thermal analysis and thermogravimetry (DTA/TG). The measurements were carried out from room temperature to 1000 °C at a heating rate of 10 °C/min under a synthetic air atmosphere.

To determine the mechanical strength of the samples, compressive strength tests were carried out using a universal testing machine, in accordance with the TS EN 843-1 standard. Prior to testing, the diameter and height of each cylindrical specimen (30 mm in diameter × 10 mm in thickness) were measured and recorded. The compressive strength (σ = F/A) was calculated based on the maximum failure load. For each group, at least three samples were tested, and the average compressive strength values along with standard deviations were reported. The obtained results were analyzed to assess the effect of sintering temperature and duration on mechanical performance.

In the final stage of the study, the newly fabricated UF-resin-added samples were also subjected to compressive testing under the same standards, aiming to enhance the mechanical strength. The results were compared with the outcomes of resin-free samples in Section 3.

### 2.3 Density and Pore Structure Measurements

The total open porosity and bulk density values of the samples were determined using the liquid displacement method based on Archimedes’ principle. Distilled water was used as the immersion medium, and measurements were conducted in accordance with ISO 5017 standard.^23^ The total porosity and bulk density were calculated using the saturation method (eqs 1 and 2):

$$\mathrm{Porosity}\left(\%\right)=\left[\left(m_{\mathrm{sat}}-m_{\mathrm{dry}}\right)/\left(m_{\mathrm{sat}}-m_{\mathrm{sub}}\right)\right]\times 100$$ 1

$$\mathrm{Density}=m_{\mathrm{dry}}/\left(m_{\mathrm{sat}}-m_{\mathrm{sub}}\right)$$ 2

where *m*_dry_ is the dry weight, *m*_sat_ is the saturated weight, and *m*_sub_ is the suspended weight in water. Measurements were performed on three samples for each sintering condition. Furthermore, the specific surface area and pore size distribution were analyzed using the BET (Brunauer–Emmett–Teller) method to quantitatively evaluate the effect of the binder on microstructural densification.^24^

## 3 Results and Discussion

### 3.1 Phase Analysis by XRD

Initially, to examine the effect of sintering temperature on β-SiC formation, samples sintered at 1350, 1400, and 1500 °C for 3 h were subjected to characterization tests. Figure 1 presents the XRD patterns of samples sintered at 1350, 1400, and 1500 °C for 3 h, illustrating the effect of sintering temperature on SiC phase formation.

**Figure 1:**
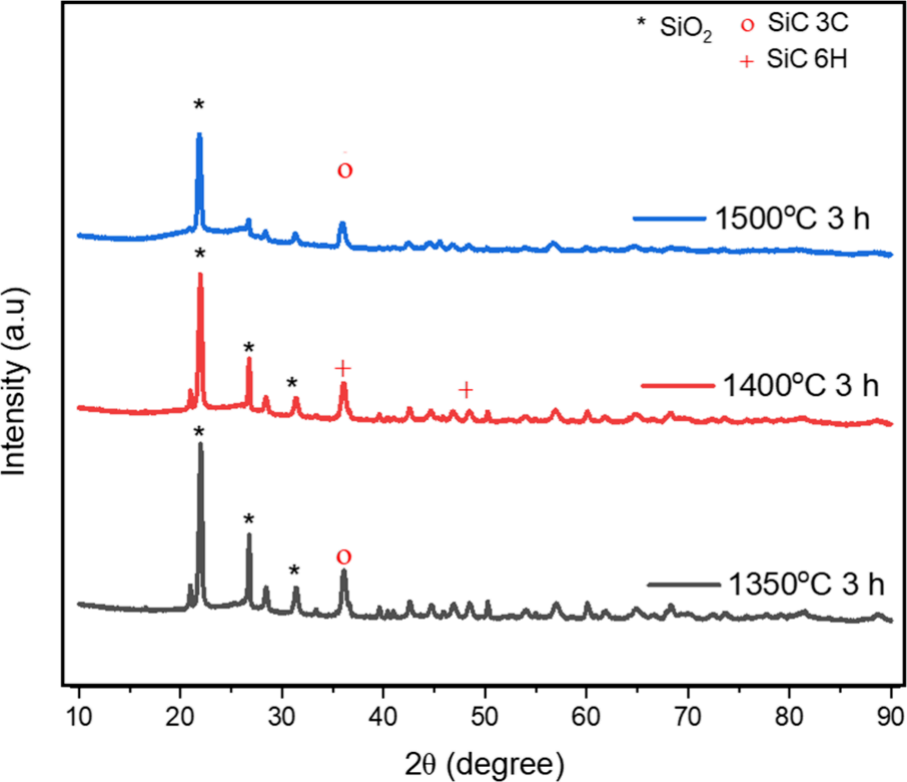
Comparison of XRD patterns of samples sintered at 1350, 1400, and 1500 °C for 3 h.

When the XRD patterns shown in Figure 1 are examined, peaks corresponding to the SiO_2_ cristobalite structure (PDF# 01-076-0940) are observed at 2θ = 22, 26.77, and 31.39° for the sample sintered at 1350 °C. Additionally, a peak at 2θ = 36.13° is attributed to the 3C cubic structure of SiC (PDF# 00-029-1129).

For the sample sintered at 1400 °C, peaks corresponding again to the SiO_2_ cristobalite structure (PDF# 01-076-0940) appear at 2θ = 21.96, 26.76, and 31.33°. Furthermore, peaks observed at 2θ = 36.03 and 48.44° are associated with the initial formation of SiC polytypes, closely matching the 6H hexagonal structure (PDF# 01-073-1663). These signals are interpreted as transitional disorder or stacking faults typical of the early stages of carbothermal reduction, as the system begins to transform toward the more stable 3C-SiC phase observed at higher temperatures.

In the sample sintered at 1500 °C, a peak at 2θ = 21.79° corresponds to SiO_2_ cristobalite (PDF# 01-076-0934), while a peak at 2θ = 26.75° is attributed to SiO_2_ quartz (PDF# 01-083-2467). A peak at 2θ = 31.33° is identified as a hexagonal SiO_2_ structure. Peaks observed at 2θ = 35.79° indicate the presence of SiC 3C cubic (PDF# 00-049-1623). Overall, Figure 1 illustrates that as the temperature increases, the characteristic peaks of the 3C-SiC phase grow more intense, while the SiO_2_ peaks progressively weaken, indicating the successful advancement of the carbothermal reduction process.

After investigating the effect of temperature on the formation of β-SiC during sintering, the influence of sintering duration was examined by comparing the XRD patterns of samples sintered at 1500 °C for 1, 3, and 6 h, as shown in Figure 2.

**Figure 2:**
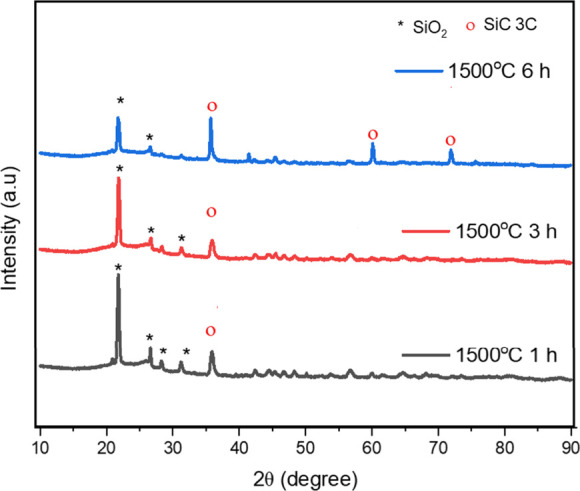
Comparison of XRD patterns of samples sintered at 1500 °C for 1, 3, and 6 h.

The XRD patterns presented in Figure 2 show that the sample sintered at 1500 °C for 1 h exhibited diffraction peaks corresponding to SiO_2_-based phases. Peaks observed at 2θ = 21.82, 28.21, and 31.19° were attributed to the cristobalite structure (PDF# 01-076-0934 and PDF# 00-003-0270), while the peak at 2θ = 26.63° corresponded to the quartz phase (PDF# 01-079-1906). In addition, diffraction peaks at 2θ = 35.79° confirmed the presence of cubic 3C-SiC (PDF# 01-075-0254).

The XRD analysis of the sample sintered at 1500 °C for 3 h is presented in detail in Figure 1. For the sample sintered at 1500 °C for 6 h, diffraction peaks observed at 2θ = 21.74, 28.48, and 31.34° were associated with cubic SiO_2_ phases (PDF# 01-076-0931 and PDF# 00-001-0438), while the peak at 2θ = 26.69° corresponded to the quartz phase (PDF# 01-085-0335). Moreover, peaks at 2θ = 35.76, 60.11, and 71.98° are attributed to the cubic 3C-SiC phase (PDF# 01-074-2307). Following the evaluation of the sintering time, the sintering temperature was increased to 1550 °C to further investigate the thermal evolution and phase stability of SiC.

The XRD patterns of the samples sintered at 1500 and 1550 °C for 6 h are compared in Figure 3. The XRD pattern analysis of the sample sintered at 1500 °C for 6 h is provided in Figure 2. In the XRD pattern of the sample sintered at 1550 °C for 6 h, a peak corresponding to the SiO_2_ cristobalite phase (PDF# 00-039-1425) is observed at 2θ = 21.76°.

**Figure 3:**
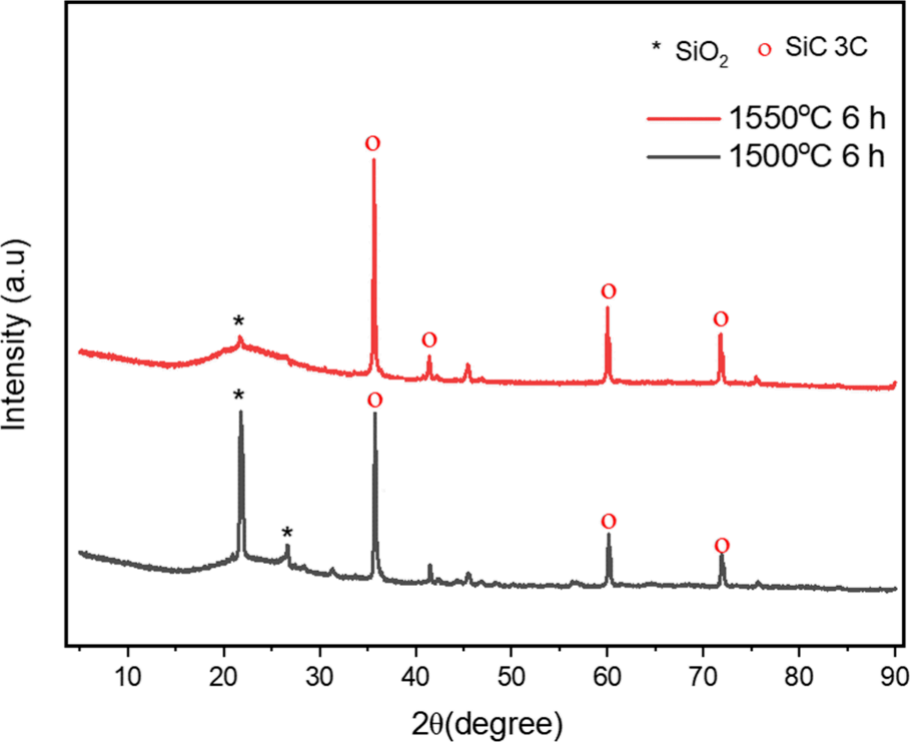
Comparison of XRD patterns of samples sintered at 1500 and 1550 °C for 6 h.

For the SiC phases, characteristic diffraction peaks are identified at 2θ = 35.66, 41.44, 45.46, 59.98, and 71.77°. The sharpening of the diffraction peaks at 1550 °C confirms that the cubic 3C-SiC structure reaches its highest degree of crystallinity under these conditions, with residual SiO_2_ levels reaching their minimum.

### 3.2 Oxidation Resistance and Thermal Stability (DTA/TG Analysis)

To evaluate the thermal stability and oxidation behavior of the synthesized samples, simultaneous TG/DTA analyses were performed on the binder-free (1500) and UF-resin-bonded (1500R) specimens. Figure 4 presents the comparative TG/DTA curves of the resin-free (1500) and resin-bonded (1500R) samples.

**Figure 4:**
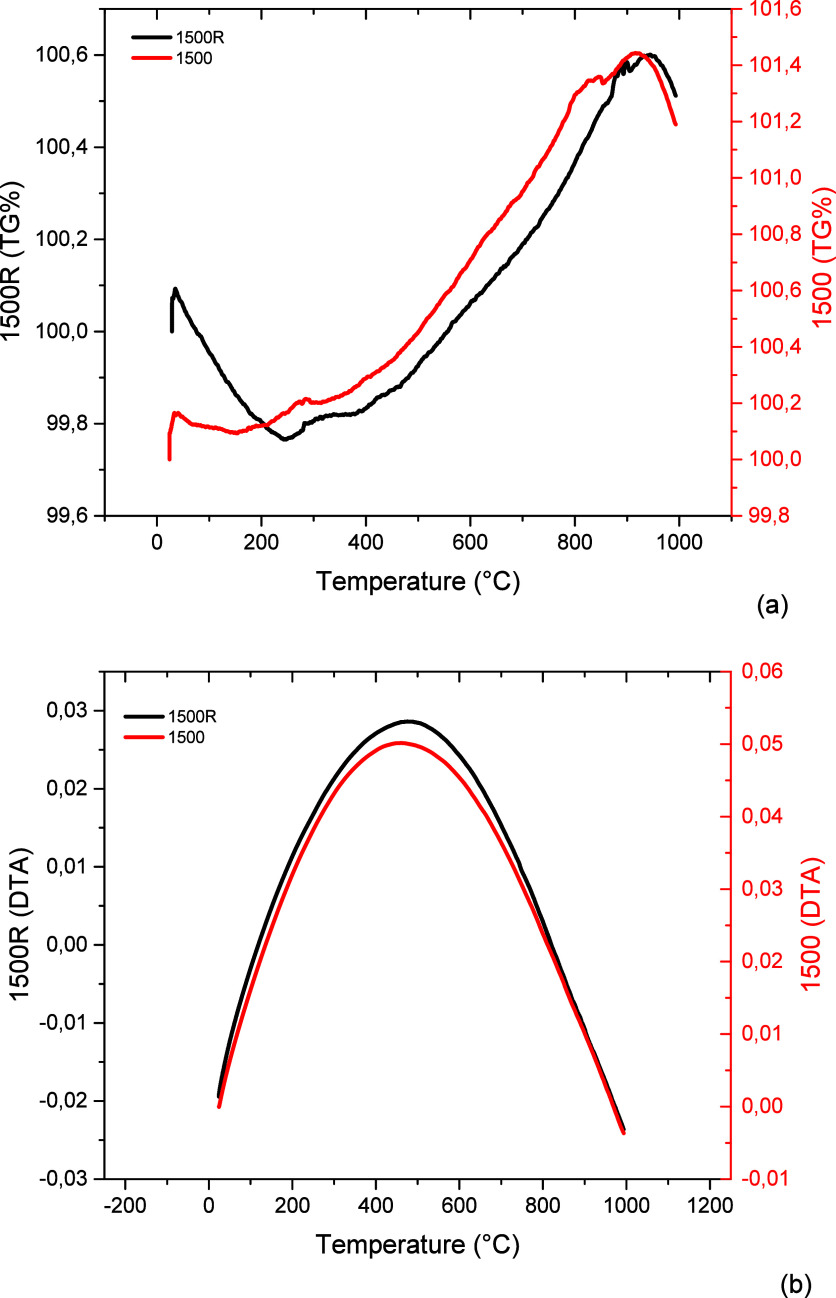
Simultaneous thermal analysis of the binder-free (1500) and resin-bonded (1500R) samples under a synthetic air atmosphere: (a) thermogravimetry (TG) curves illustrating mass change and oxidation stability, and (b) differential thermal analysis (DTA) curves showing the corresponding exothermic heat flow.

As observed in the TG curves (Figure 4a), the resin-bonded sample exhibited relatively stable thermal behavior with minimal cubic mass change up to 1000 °C. In contrast, the binder-free sample showed a distinct mass gain. This behavior corresponds to the ″passive oxidation″ mechanism, where a silica layer forms on the surface, which is further supported by the broad exothermic peak in the DTA curve (Figure 4b). The absence of mass loss confirms that the hybrid insulation system significantly suppressed active oxidation (gasification). Furthermore, the enhanced thermal stability of the 1500R sample suggests that the UF-resin-derived carbon skeleton may act as a partially protective carbon-rich barrier at elevated temperatures.

### 3.3 Microstructural Evolution (SEM Analysis)

SEM analyses were carried out to examine the effect of binder addition on the microstructural evolution and pore morphology of the synthesized SiC samples. Representative SEM images obtained at different magnifications are shown in Figure 5.

**Figure 5:**
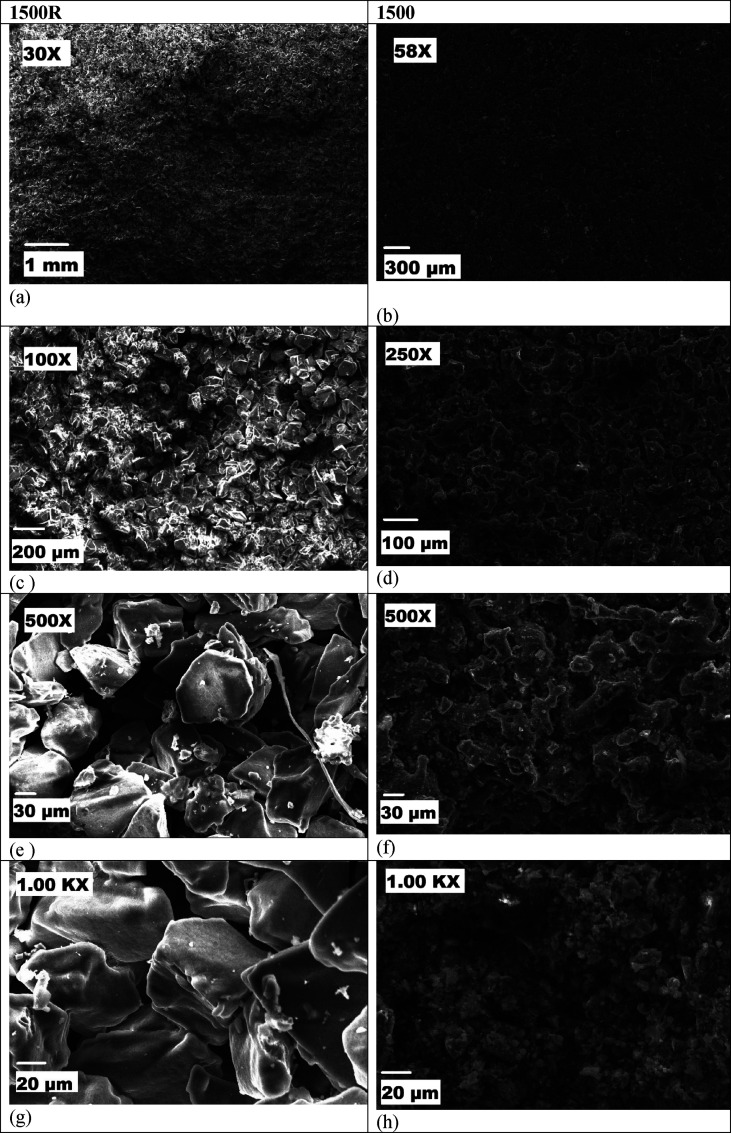
SEM images of the binder-free (b, d, f, h) and UF-resin-bonded (a, c, e, g) SiC samples at different magnifications. The binder-free samples exhibit interconnected porous structures and limited neck formation, whereas the UF-resin-bonded samples show a denser morphology with improved intergranular bonding. Scale bars are indicated in each image.

The images reveal a distinct difference in morphology driven by the binder. The resin-free sample (1500) shows a porous surface with initial necking stages. Conversely, the resin-bonded sample (1500R), examined from fracture surface particles, reveals sharp, angular cleavage planes. This morphology indicates a brittle fracture mode typical of dense ceramics, suggesting that the resin-derived carbon skeleton promoted stronger intergranular bonding compared to the loosely sintered binder-free samples.

### 3.4 Densification, True Density, and Pore Structure Evolution

To comprehensively evaluate the microstructural evolution and the role of the binder, bulk density (Archimedes method), apparent porosity, specific surface area, and total pore volume (BET) were analyzed (Table 2).

**Table 2:** Densification and Pore Structure Parameters of the Synthesized SiC Samples

sample (°C)	apparent porosity (%)	**bulk density** **(g/cm** **^3^** **)**	**specific surface area** **(m** **^2^** **/g)**	**total pore volume** **(cm** **^3^** **/g)**
**1350**	61.25	2.45	8.71	0.0267
**1400**	64.53	2.44	58.85	0.0621
**1500 (binder-free)**	66.88	2.03	33.94	0.0359
**1500R (resin-bonded)**			0.35	0.00038
**1550**	70.93	1.94	<0.1	0.0012

As the sintering temperature increased from 1350 to 1550 °C in binder-free samples, the Archimedes measurements revealed a progressive decrease in bulk density alongside an increase in apparent porosity. This macroscopic structural expansion is fundamentally driven by the active carbothermal reduction reaction (SiO_2_ + 3C →SiC + 2CO), where the rapid evolution of gaseous byproducts creates an extensive internal network of void channels. This interpretation is strongly supported by the BET results; at 1400 °C, the specific surface area experienced a dramatic surge to 58.85 m^2^/g with a total pore volume of 0.0621 cm^3^/g. To further investigate the evolution of the pore structure, nitrogen adsorption–desorption isotherms were obtained for all representative samples. The adsorption behavior provides additional insight into the influence of sintering temperature and UF resin addition on pore accessibility and microstructural densification. The corresponding adsorption–desorption isotherms are presented in Figure 6.

**Figure 6:**
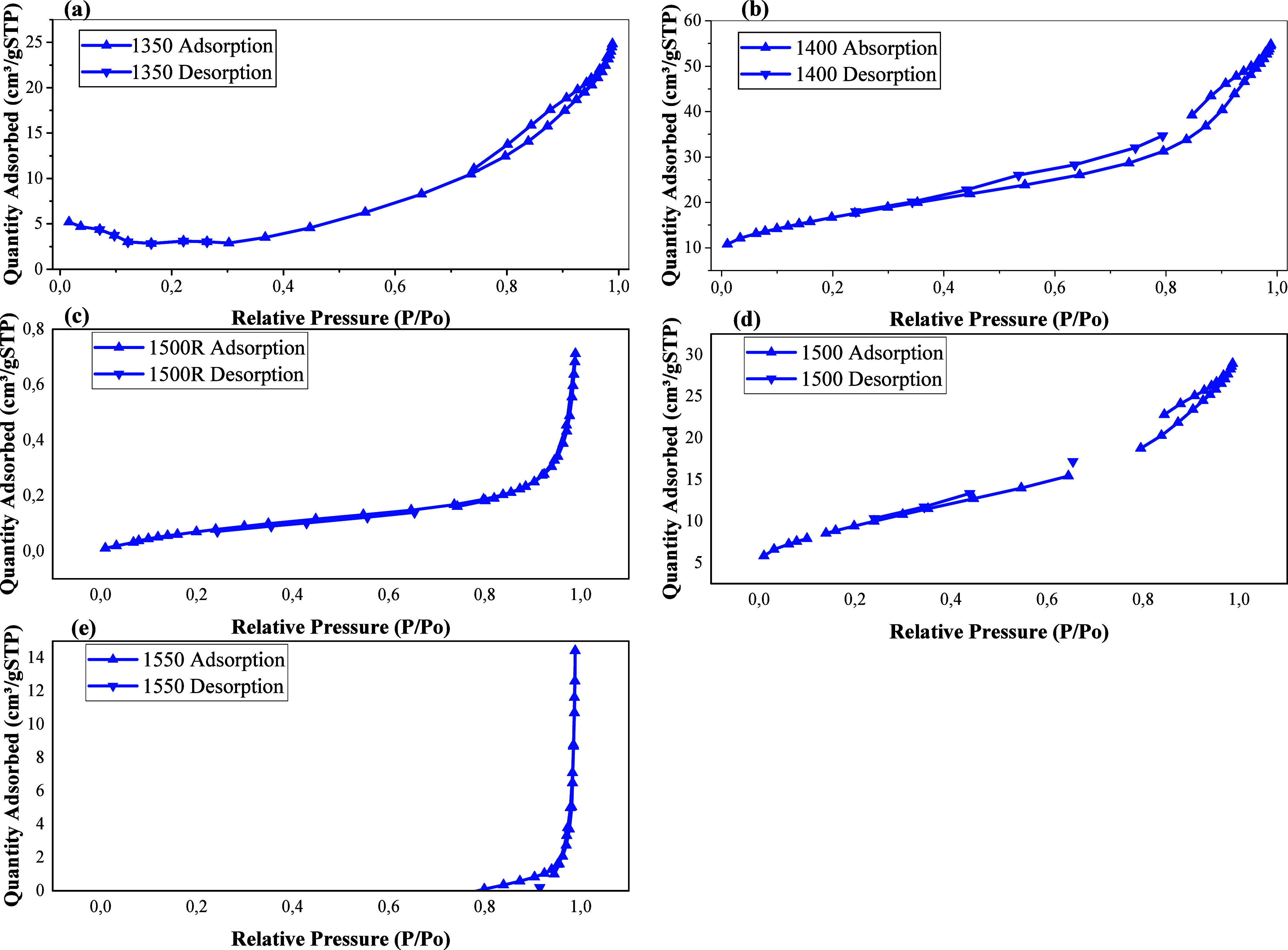
Nitrogen adsorption–desorption isotherms of the SiC samples sintered under different conditions: (a) 1350 °C, (b) 1400 °C, (c) 1500 °C (binder-free), (d) 1500R (resin-bonded), and (e) 1550 °C.

While the binder-free samples maintained a complex porous network even at 1500 °C (33.94 m^2^/g), the addition of UF resin significantly altered the densification behavior. For the 1500R sample, traditional Archimedes measurements were not applicable due to the extensive sealing of the open pore networks, which prevents water infiltration. Instead, its substantial densification was strongly supported by the low specific surface area values (0.35 m^2^/g) and minimal pore volume (0.00038 cm^3^/g). This indicates that the resin-derived glassy carbon skeleton likely contributes to the reduction of accessible open porosity and induces intrinsic grain-boundary densification, directly correlating with the significant mechanical strength enhancement.

Although the 1550 °C sample exhibited very low BET surface area values, the Archimedes measurements still indicated high apparent porosity. This difference is likely associated with the formation of large macropores that contribute significantly to bulk porosity but contribute less to the specific surface area measured by nitrogen adsorption.

### 3.5 Compressive Strength Test Results

Figure 7 shows the compressive strength of SiC samples fabricated without resin under different sintering temperatures and durations.

**Figure 7:**
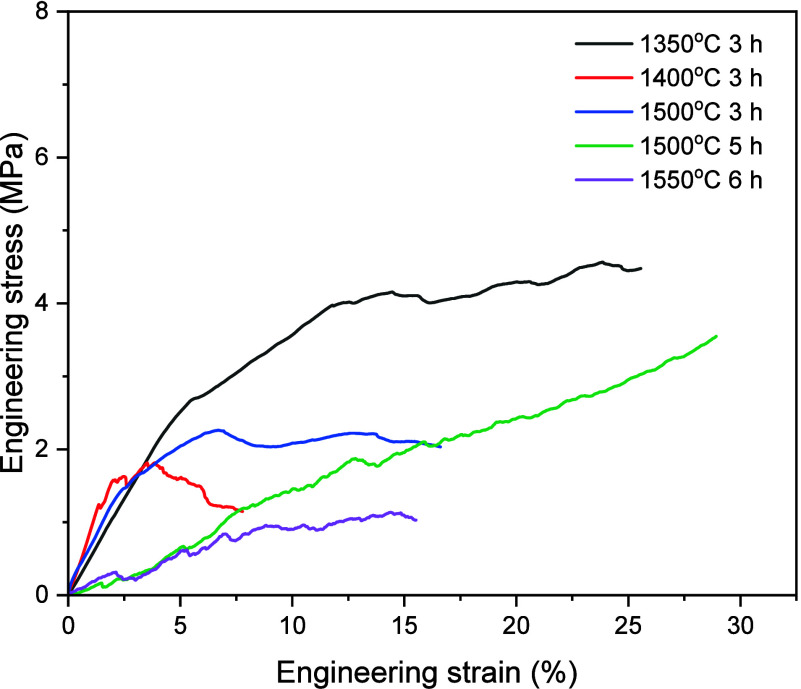
Compressive strength graphs of SiC samples fabricated without resin under different sintering temperatures and durations.

According to the compressive strength test results in Figure 7, the sample sintered at 1350 °C for 3 h exhibited the highest strength, with a maximum compressive stress of 4.5 MPa and a strain of approximately 25%. After sintering at 1400 °C for 3 h, the maximum stress decreased to 1.9 MPa, and the strain dropped to 6%. Partial recovery was observed in the samples sintered at 1500 °C for 3–6 h; maximum compressive stress increased to the range of 2.0–2.25 MPa, while strain values were recorded between 14 and 16%. However, for the sample sintered at 1550 °C for 6 h, a noticeable increase in porosity resulted in a reduced maximum compressive stress of 1.15 MPa and a strain of 14%. This behavior is attributed to the dual effects of elevated temperature and prolonged sintering on the microstructure. As the temperature increases, the phase transformation of SiO_2_ accelerates, promoting the formation of SiC. Extended treatment at 1500 °C appears to provide a balance between grain growth and bonding density, thereby resulting in partial densification and improved strength and deformation parameters compared to those at 1400 °C. The fluctuations observed in the stress–strain curves are associated with the compaction of porous structures under load and the occurrence of sudden microcracks.

It is evident that the mechanical strength of resin-free samples remains limited depending on the sintering temperature and duration. Therefore, to reduce porosity and enhance intergranular bonding, the binder type was modified, and urea–formaldehyde (UF) resin was employed. Figure 8 presents the compressive strength results of these resin-containing samples, tested under the same conditions.

**Figure 8:**
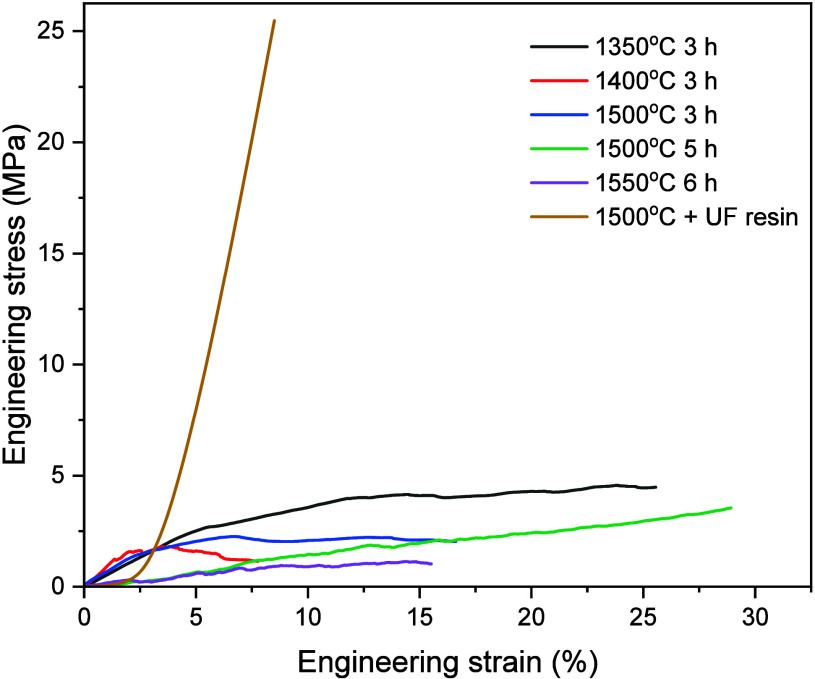
Comparison of compressive strength results for SiC samples with and without resin.

As shown in Figure 8, the SiC sample containing resin reached a maximum compressive strength of 25.5 MPa with a strain of 8.5% after only 15 min of sintering at 1500 °C. Compared to resin-free samples, a mechanical strength approximately four to five times higher was achieved. Furthermore, the reduction in porosity limited the occurrence of sudden microcracks under applied load, which is reflected in the significantly smoother stress–strain curve. These results indicate that the binder type plays a critical role not only in shaping but also in determining the postsintering mechanical performance of the samples.

## 4 Discussion

In this study, it was observed that sintering temperature and duration under atmospheric conditions had a significant influence on phase formation, microstructure, and mechanical properties.

XRD analyses revealed an increased crystallization of β-SiC polytypes with rising temperature and time. Particularly at 1500 and 1550 °C, the peak intensities and matching scores for the 3C-SiC phase reached notable levels. The proposed atmospheric route not only prevents macro-oxidation through localized getter reactions but also facilitates thermodynamic pathways conducive to phase boundary nucleation and structural alignment, as conceptually observed in the controlled crystallization and anisotropic growth of advanced layered materials under tailored thermal environments.^25–28^ However, under the same conditions, increased porosity and water absorption limited the mechanical strength, despite the presence of the desired phase formation.

As evidenced by the mechanical testing results, the structural integrity of binder-free samples was severely compromised at elevated temperatures. This reduction in strength is associated with grain growth and pore formation due to oxidation. However, porosity evolution is not solely driven by oxidation. The carbothermal reduction reaction itself (SiO_2_ + 3C → SiC + 2CO) generates significant amounts of CO gas. In pressureless sintering, the release of these gaseous byproducts creates void channels, contributing to the observed porosity increase.

The microstructural degradation observed at elevated temperatures was further elucidated through high-resolution SEM analysis, providing better clarity on pore morphology. As shown in the SEM micrographs, the binder-free samples exhibited a highly interconnected void network, whereas the UF-resin bonded samples (1500R) displayed a significantly more integrated and dense structure. This densification is quantitatively supported by the BET results, where the total pore volume was reduced to near-zero levels (0.00038 cm^3^/g). The resin-derived glassy carbon skeleton acts as a reactive bridge, contributing to the sealing of open channels created by CO gas evolution during the carbothermal reduction process.

Consequently, the introduction of UF resin fundamentally transformed the densification mechanism.^29,30^ The significant improvement in mechanical strength achieved—surpassing the binder-free counterparts by 4-fold—is not merely a result of shorter sintering time but is also likely associated with the formation of a resin-derived carbon-rich network that effectively bridges the grains and seals the gas-induced porosity.^31^

In the literature, conventional Acheson processes or sintering under inert atmospheres typically require temperatures above 2000 °C, making these routes highly energy- and cost-intensive.^6–9^ Mas’udah et al. reported that β-SiC phases could be obtained from SiO_2_–C mixtures under lower sintering temperatures, although high porosity limited mechanical performance.^19^ In contrast, our study demonstrated that similar phase formations could be achieved within the 1350–1550 °C range under atmospheric conditions using a hybrid graphite–mullite insulation system. Furthermore, binder addition significantly increased microstructural densification.

Ren et al. produced porous SiC using silicon kerf waste under Ar atmosphere and reported porosity levels exceeding 60% at 1500 °C.^16^ Compared to this, our binder-assisted samples sintered under atmospheric conditions showed significantly reduced accessible open porosity and a 4–5-fold increase in mechanical strength.

Although Hidayat et al. achieved enhanced phase purity in β-SiC production via the magnesiothermic reduction method, their process required both an inert atmosphere and additional reactants.^13^ Additionally, the magnesiothermic route may lead to finer powder morphology and different reaction kinetics compared to the gas-evolving carbothermal reduction mechanism observed in the present study. In contrast, the atmospheric sintering technique employed in this study enabled β-SiC phase formation without any controlled environment or extra reactants, making the process more economical and potentially environmentally advantageous.

Lavrenchuk et al. used CO/CO_2_ gas control in an atmospheric-pressure arc reactor to minimize oxidation during SiC synthesis. Comparatively, this study demonstrated a simpler and more cost-effective approach by utilizing sacrificial carbon layers and graphite insulation, which provided a comparable oxidation control mechanism.^11^

The results are consistent with phase transformation trends reported in the literature for inert-atmosphere sintering methods: increased crystallinity at elevated temperatures, but microstructural integrity can deteriorate with prolonged sintering. More importantly, this study clearly demonstrates the critical role of binder addition in enhancing both phase formation and mechanical properties.

This work confirms that β-SiC can be synthesized from SiO_2_ and activated carbon mixtures via sintering under atmospheric conditions at relatively low temperatures. Compared to conventional high-temperature routes, this approach may reduce energy consumption. The use of a hybrid graphite–mullite insulation system and sacrificial carbon layers limited oxidation to some extent and supported phase formation. Moreover, the binder type was found to play a crucial role in determining mechanical strength and microstructural compactness. Specifically, urea-formaldehyde resin reduced porosity and provided a denser structure, resulting in compressive strength values four to five times higher. The mechanism of UF resin-induced strengthening is attributed to ″char formation″. Upon thermal decomposition, the resin forms a glassy carbon network at the grain boundaries. The gradual thermal decomposition of the UF resin during heating is also believed to contribute to localized neck formation and enhanced interparticle bonding during atmospheric sintering. Similar char-assisted densification mechanisms have been reported in carbon-containing ceramic systems, where residual carbon phases improve particle connectivity and reduce accessible open porosity.^31,32^ This rigid carbon skeleton not only restricts grain sliding but also provides a secondary, highly reactive carbon source for residual SiO_2_, promoting localized neck formation. This explains the 4-fold strength increase achieved in a short sintering time.

However, complete prevention of oxidation remains a challenge under atmospheric sintering conditions, and increased porosity with prolonged sintering is a major factor limiting mechanical properties. Limitations of this study include the inability to fully control phase purity and the relatively lower microstructural homogeneity compared to sintering under inert atmospheres.

Future studies could investigate the effect of different binder systems to further improve microstructural density and phase purity. Additionally, the optimization of hybrid insulation materials and carbon-supported reactive barriers could help minimize oxidation in atmospheric sintering. This method offers promising potential as a cost-effective and technically viable alternative for SiC production, demonstrating a practical route for scalable ceramic manufacturing under atmospheric conditions.

Despite the reduced processing temperature and elimination of inert gas systems, the present atmospheric sintering approach still involves sacrificial carbon consumption and CO/CO_2_ generation during carbothermal reduction. Therefore, the environmental advantages of the process should be interpreted cautiously. A complete quantitative evaluation of energy consumption, carbon emissions, and graphite utilization efficiency was beyond the scope of this proof-of-concept study. Future work should include detailed life-cycle assessment (LCA) and comparative environmental analysis against conventional SiC production routes such as the Acheson process.

## 5 Conclusions

This study demonstrated a binder-assisted atmospheric sintering approach for β-SiC formation from SiO_2_ and activated carbon. The developed hybrid graphite–mullite insulation system helped limit oxidation during atmospheric sintering, enabling the synthesis of β-SiC without requiring any inert or vacuum atmosphere. The binder type was found to play a decisive role in enhancing densification and mechanical performance, providing an effective control parameter for optimizing SiC microstructure.

The findings revealed that increasing both sintering temperature and time promoted β-SiC phase formation but also led to enhanced porosity, which reduced mechanical strength. Incorporation of urea–formaldehyde resin resulted in a significant improvement in densification and mechanical strength, achieving a compressive strength of 25.5 MPa within only 15 min of sintering at 1500 °C. This improved mechanical performance was directly associated with enhanced microstructural densification. BET analysis confirmed a significant reduction in both the specific surface area (0.35 m^2^/g) and total pore volume (0.00038 cm^3^/g).

In summary, this study demonstrates a binder-assisted atmospheric route that aligns with the development of more sustainable ceramic processing approaches by potentially reducing energy consumption and simplifying furnace design. The combination of binder chemistry and hybrid insulation design offers a new route toward scalable, low-cost SiC production. The findings suggest that this approach can be adapted for industrial-scale synthesis and may inspire further development of potentially environmentally advantageous sintering technologies for advanced ceramics.

## References

[1] Wu R., Zhou K., Yue C. Y., Wei J., Pan Y. (2015). Recent progress
in synthesis, properties and potential applications of SiC nanomaterials. Prog. Mater. Sci..

[2] Sun K., Wang T., Gong W., Lu W., He X., Eddings E. G., Fan M. (2022). Synthesis and potential applications
of silicon carbide nanomaterials/nanocomposites. Ceram. Int..

[3] Lamon, J. , 2020. Properties and characteristics of SiC and SiC/SiC composites. In: Konings, R. J. M. , Stoller, R. E. (Eds.), Comprehensive Nuclear Materials (2nd ed.). Elsevier, Oxford, pp 400–418. 10.1016/B978-0-12-803581-8.11717-5.

[4] Hinoki T., Kohyama A. (2005). Current status of SiC/SiC composites
for nuclear applications. Ann. Chim. –
Sci. Matér..

[5] Snead L. L., Nozawa T., Katoh Y., Byun T. S., Kondo S., Petti D. (2007). Handbook of SiC properties
for fuel performance modeling. J. Nucl. Mater..

[6] Guichelaar, P. J. Acheson process. In: Weimer, A. W. (Ed.), Carbide, Nitride and Boride Materials Synthesis and Processing. Springer Netherlands, Dordrecht, 1997. pp 115–129. 10.1007/978-94-009-0071-4_4.

[7] Acheson E. G. (1893). Carborundum:
Its history, manufacture and uses. Journal of
the Franklin Institute.

[8] Mühlbauer A., Keiner D., Galimova T., Breyer C. (2024). Analysis of production
routes for silicon carbide using air as carbon source empowering negative
emissions. Mitigation and Adaptation Strategies
for Global Change.

[9] Knigawka P. A., Ganczewski G. J. (2023). Environmental assessment of hard
coal char as a carbon
reductant for silicon alloys production. International
Journal of Life Cycle Assessment.

[10] Sokolowski K., Blazewicz S., Marzec M. M., Bernasik A., Fraczek-Szczypta A. (2024). Synthesis
and growth mechanism of silicon-based nanostructures from polysiloxane
via CVD process. Surf. Coat. Technol..

[11] Lavrenchuk A. A., Speranskiy M.Yu., Pak A. Y., Korchagina A. P., Vlasov A. V. (2024). Synthesis of silicon
carbide using an AC atmospheric-pressure
arc reactor. J. Alloys Compd..

[12] Colombo P., Mera G., Riedel R., Sorarù G. D. (2010). Polymer-
Derived Ceramics: 40 Years of Research and Innovation in Advanced
Ceramics. J. Am. Ceram. Soc..

[13] Hidayat, N. ; Ardiansyah; Fuad, A. ; Mufti, N. ; Amalia Fibriyanti, A. ; Insjaf Yogihati, C. ; Prihandoko, B. Magnesiothermic reduction synthesis of silicon carbide with varying temperatures: Structural and mechanical features. IOP Conference Series: Materials Science and Engineering; IOP Publishing 2019, 515, 012079DOI: 10.1088/1757-899X/515/1/012079.

[14] Kim H. M., Kim Y. W. (2019). Low temperature pressureless sintering
of silicon carbide
ceramics with alumina–yttria–magnesia–calcia. J. Ceram. Soc. Jpn..

[15] Perthuis H., Colomban Ph. (1984). Well densified Nasicon
type ceramics elaborated using
sol-gel process and sintering at low temperatures. Mater. Res. Bull..

[16] Ren X., Ma B., Qian F., Yang W., Liu G., Zhang Y., Yu J., Zhu Q. (2021). Green synthesis of porous SiC ceramics using silicon
kerf waste in different sintering atmospheres and pore structure optimization. Ceram. Int..

[17] Sizyakov V. M., Bazhin V.Yu., Piirainen V.Yu., Sharikov F.Yu., Mas’ko O. N. (2023). Implementation
of self-propagating low-temperature synthesis to produce pure silicon
carbide. Refractories and Industrial Ceramics.

[18] Stutz D., Prochazka S., Lorenz J. (1985). Sintering and microstructure formation
of silicon carbide. J. Am. Ceram. Soc..

[19] Mas’udah K. W., Diantoro M., Fuad A. (2018). Synthesis and structural analysis
of silicon carbide from silica rice husk and activated carbon using
solid-state reaction. J. Phys.:Conf. Ser..

[20] Soldati R., Zanelli C., Cavani G., Battaglioli L., Guarini G., Dondi M. (2022). Improving the sustainability
of ceramic
tile-making by mixing spray-dried and dry-granulated powders. Boletín de la Sociedad Española de Cerámica
y Vidrio.

[21] Freedman, M. R. , Millard, M. L. , 1986. Improved consolidation of silicon carbide. In: 10th Annual Conference on Composites and Advanced Ceramic Materials: Ceramic Engineering and Science Proceedings. John Wiley & Sons, Ltd., pp 884–892. 10.1002/9780470320341.ch15.

[22] Cullity, B. D. ; Stock, S. R. , Elements of X-ray Diffraction. 3rd ed. Prentice Hall: Upper Saddle River, NJ, 2001. ISBN: 9780201610918.

[23] ISO , 2013. ISO 5017: Dense shaped refractory products — Determination of bulk density, apparent porosity and true porosity. ISO , Geneva. Available at: https://www.iso.org/obp/ui/en/#iso:std:iso:5017:ed-3:v1:en (Accessed 25 July 2025).

[24] Brunauer S., Emmett P. H., Teller E. (1938). Adsorption
of gases in multimolecular
layers. J. Am. Chem. Soc..

[25] Xu Y., Shi X., Zhang Y., Zhang H., Zhang Q., Huang Z., Xu X., Guo J., Zhang H., Sun L., Zeng Z., Pan A., Zhang K. (2020). Epitaxial nucleation and lateral growth of
high-crystalline black phosphorus films on silicon. Nat. Commun..

[26] Li X. (2021). High-performance polarization-sensitive
photodetectors on two-dimensional
β-InSe. Natl. Sci. Rev..

[27] Xue T., Liang W., Li Y., Sun Y., Xiang Y., Zhang Y., Dai Z., Duo Y., Wu L., Qi K., Shivananju B. N., Zhang L., Cui X., Zhang H., Bao Q. (2019). Ultrasensitive detection
of miRNA with an antimonene-based surface plasmon resonance sensor. Nat. Commun..

[28] Zhao J., Zhu J., Cao R., Wang H., Guo Z., Sang D. K., Tang J., Fan D., Li J., Zhang H. (2019). Anisotropic two-dimensional
atomic layered black phosphorus. Nat. Commun..

[29] Colombo P., Mera G., Riedel R., Sorarù G. D. (2010). Polymer-derived
ceramics: 40 years of research and innovation in advanced ceramics. J. Am. Ceram. Soc..

[30] Eom J. H., Kim Y. W., Song I. H. (2013). Processing
and properties of macroporous
silicon carbide ceramics: A review. J. Asian
Ceram. Soc..

[31] Kozekanan B. S., Moradkhani A., Baharvandi H., Ehsani N. (2021). Mechanical properties
of SiC-C-B4C composites with different carbon additives produced by
pressureless sintering. Int. J. Appl. Ceram.
Technol..

[32] Tang Y., Zhou X., Zhang Q., Chen L., Zhao K., Wu Z. (2022). Effect of resin pyrolysis
behavior on the pore structure and mechanical
properties of porous silicon carbide ceramics. Ceram. Int..

